# Impact of chronic graft-versus-host-disease on intensive care outcome in allogeneic hematopoietic stem cell recipients

**DOI:** 10.1038/s41409-022-01875-4

**Published:** 2022-12-10

**Authors:** Catherina Lueck, Asterios Tzalavras, Philipp Wohlfarth, Elisabeth Meedt, Michael Kiehl, Amin T. Turki, Marius M. Hoeper, Matthias Eder, Julia Cserna, Nina Buchtele, Daniel Wolff, Peter Schellongowski, Gernot Beutel, Tobias Liebregts

**Affiliations:** 1 Department for Hematology, Hemostasis, Oncology and Stem Cell Transplantation, Hannover Medical School, Hannover, Germany; 2 iCHOP – Intensive Care in Hematologic and Oncologic Patients, Essen, Germany; 3 Department of Hematology and Stem Cell Transplantation, West German Cancer Center, University of Duisburg-Essen, University Hospital Essen, Essen, Germany; 4 Department of Medicine I, Medical University of Vienna, Vienna, Austria; 5 Department of Internal Medicine III, Hematology and Oncology, University Hospital Regensburg, Regensburg, Germany; 6 Department of Internal Medicine I, Clinic Frankfurt/Oder GmbH, Frankfurt/Oder, Germany; 7 Department for Respiratory Diseases and German Centre of Lung Research (DZL), Hannover Medical School, Hannover, Germany

**Keywords:** Risk factors, Stem-cell research, Prognosis

## Abstract

Chronic graft-vs-host-disease (cGvHD) is the most relevant long-term complication after allogeneic stem cell transplantation (HSCT) with major impact on non-relapse mortality, but data on intensive care unit (ICU) outcome are missing. In this retrospective, multicenter study we analyzed 174 adult HSCT recipients with cGvHD requiring intensive care treatment. Skin, pulmonary, liver, and intestinal involvement were present in 76.7%, 47.1%, 38.1% and 24.1%, respectively, and a total of 63.2% had severe cGvHD. Main reasons for ICU admission were respiratory failure (69.7%) and sepsis (34.3%). Hospital- and 3-year OS rates were 51.7% and 28.6%, respectively. Global severity of cGvHD did not impact short- and long-term survival. However, patients with severe liver cGvHD or the overlap subtype had a reduced hospital survival, while severe pulmonary cGvHD was associated with worse long-term survival. In multivariate analysis need for invasive ventilation (HR 1.08 (95% CI 1.02–1.14)) or hemodialysis (HR 1.73 (95% CI 1.14–2.62)) and <1 year since HSCT (HR 1.56 (95% CI 1.03–2.39)) were independently associated with a poorer survival. While the global severity of cGvHD does not per se affect patients’ survival after intensive care treatment, pre-existing severe hepatic, intestinal or pulmonary cGvHD is associated with worse outcomes.

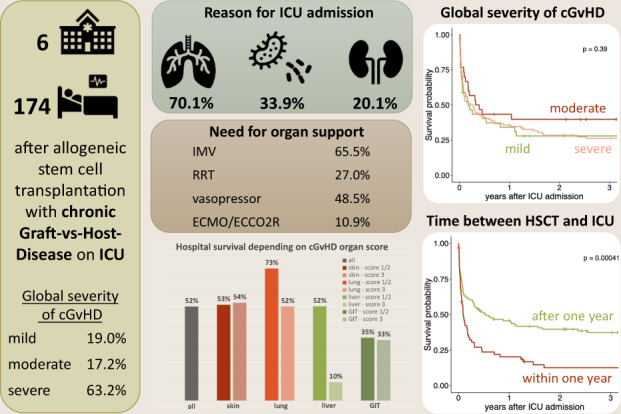

## Introduction

With the increasing frequency of allogeneic hematopoietic stem cell transplantations (HSCT) performed annually [1], the number of long-term survivors is also increasing. Chronic graft-vs-host-disease (cGvHD) is the most relevant long-term complication after HSCT affecting approximately 50% of all patients with a major impact on morbidity and non-relapse mortality [2]. This is primarily related to the dysfunction of the affected organ systems and immunodeficiency caused by cGvHD itself and the required immunosuppressive treatment. Both increase the risk of acute organ dysfunction or deterioration and the resulting demand for intensive care in this patient population. In addition to known risk factors of intensive care unit (ICU) survival, such as the number of organ failures and the need for organ support (invasive mechanical ventilation (IMV), vasopressors, hemodialysis), the presence of active acute graft-vs-host-disease is a known risk factor for poor survival in HSCT recipients [3–6]. In general, patients with severe cGvHD have a higher non-relapse mortality. According to a recent observational single-center study, patients with severe cGvHD and pulmonary, hepatic and/or gastrointestinal (GI) involvement have a significantly higher mortality compared to patients with severe cGvHD without corresponding organ involvement [7]. Of notice, there was no significant difference in overall survival (OS) in patients with mild to moderate cGvHD compared to patients without cGvHD. Other studies have reported reduced survival rates in patients with severe cGvHD compared to mild/moderate cases [2, 8]. In addition to the organs affected, the clinical course also appears to be prognostically relevant: patients with a progressive subtype emerged from acute GvHD [7] and patients with an overlap subtype with simultaneous features of both chronic and acute GvHD [9] have shorter survival rates.

However, no data are available on the intensive care outcome in HSCT recipients with cGvHD. Within the iCHOP (Intensive Care in Hematologic and Oncologic Patients) initiative we therefore aimed to investigate both short- and long-term outcomes of critically ill HSCT recipients with cGvHD to support decision making for ICU management.

## Patients and methods

### Patients and data collection

We retrospectively analyzed patients with cGvHD after allogeneic HSCT, who were admitted to ICU in one of the five study centers in Germany or Austria (University Hospital Essen [*n* = 68], Hannover Medical School [*n* = 63], Medical University Vienna [*n* = 31], University Hospital Regensburg [*n* = 7], Clinic Frankfurt/Oder GmbH [*n* = 6]). All centers are well experienced in treatment of critically ill HSCT recipients.

Patients transplanted between January 2000 and October 2018, who were admitted to ICU until October 2019 with diagnosed cGvHD were included. Data collection included demographics, underlying hematologic disease, transplant-associated data as well as organ subtype and scores of cGvHD at the time of ICU admission. Diagnosis of cGvHD was based on clinical and / or laboratory values according to NIH consensus criteria 2005 [10]. In patients diagnosed prior to publication of the consensus criteria in 2005, the cGvHD severity was retrospectively assessed from hospital records. Patients, who were classified as late acute GvHD, were excluded from the analysis. Organ scoring was available for lung, skin, liver and GI manifestations of cGvHD for all patients. Scoring for eye, mouth and fascia involvement was not available in full detail. Therefore, we only report the occurrence and combined these patients as “other subtypes” in the Cox regression analysis. The organ scores were summarized to provide a global severity score of mild, moderate, or severe cGvHD [10]. The cGvHD subcategories *classic* and *overlap* were not clearly documented in most patient records. Nevertheless, to perform an appropriate subgroup analysis, patients with GI and / or liver involvement with variable skin involvement, but without involvement of the lung or other solely cGvHD-typical organs were categorized as *overlap*.

The following ICU-related data were collected: reasons for ICU admission, interval between HSCT and ICU admission, duration of ICU and hospital stay, and the use of life sustaining therapies (IMV, non-invasive ventilation (NIV), renal replacement therapy (RRT), use of catecholamines in the first 24 h). Sepsis was defined according to The Third International Consensus Definitions for Sepsis and Septic Shock (Sepsis-3) [11]. The severity of illness at ICU admission was assessed using the Simplified Acute Physiology Score II (SAPS II) [12], the Acute Physiology And Chronic Health Evaluation (APACHE) II score [13], and the Sequential Organ Failure Assessment (SOFA) score [14]. Survival status was recorded at ICU and hospital discharge as well as 12 months after ICU admission and last time of follow-up.

### Statistical analysis

Median and interquartile range (IQR) were used to describe continuous variables, numbers, and percentages for categorial variables. The Mann–Whitney U test was used to compare continuous data; a Pearson’s *χ*^2^ test was used for categorical data including ICU and hospital survival. ICU survival was defined as transfer from ICU to normal ward or rehabilitation hospital. The time of discharge from acute inpatient treatment defined hospital survival. Overall survival was calculated from day of ICU admission until death and was censored for surviving patients at the time of last follow-up. One-year and OS after ICU admission were estimated by the Kaplan–Meier method, and a stratified log-rank test was used to calculate differences between groups.

Hazard ratios and 95% confidence intervals for potential prognostic factors of OS were calculated using univariate and multivariate Cox regression models. The variables *need for ventilation* and *need for vasopressors within 24* *h of ICU admission* were classified as time-dependent variables. Collinearity was excluded.

All statistical tests were two-sided with a required significance level of <0.05. Statistical analysis was performed using R software (version 4.0.0 for Mac, packages *survival, survminer*). Graphics were created using R (package *ggplot2*).

## Results

### Patient characteristics

**Fig. 1:**
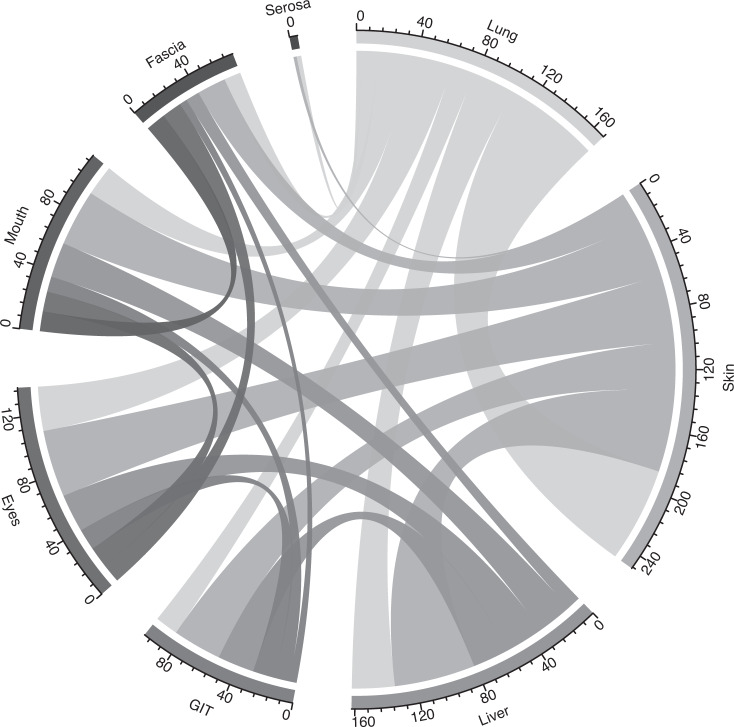
Frequency of organ-specific subtype combination. Chord diagram presenting the frequency of individual combinations of organ-specific subtypes. The size of the link is defined as the absolute value of the organ combination.

**Fig. 2:**
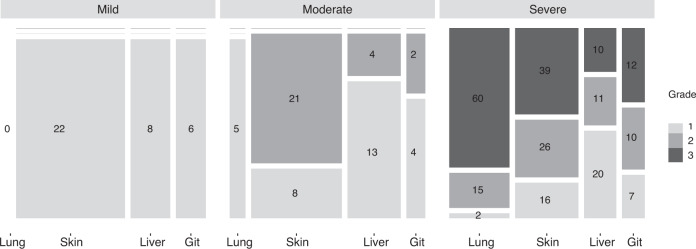
Organ scoring in global severity categories of cGvHD (mild = 33, moderate = 30, severe = 110). The mosaic diagram shows the composition of cGvHD global severity subgroups mild, moderate, and severe with the absolute number of patients for each organ score of lung, skin, liver, and gastrointestinal tract (GIT) manifestations (Scores 0–3).

A total of 174 patients with cGvHD at time of ICU admission were identified, whose characteristics are detailed in Table 1. HSCT was performed from unrelated donors and with HLA-mismatch in 112 (64.7%) and 28 (16.2%) patients, respectively. Regarding cGvHD, skin and pulmonary involvement was present in 132 (76.7%) and 82 (47.1%) patients, respectively. Criteria for cGvHD of the liver and the GI tract were fulfilled in 66 (38.1%) and 41 (24.1%) HSCT recipients. Involvement of eyes, mouth, fascia, and serosa were documented for 51 (29.3%), 46 (26.4%), 19 (10.9%) and 4 (2.3%) patients, respectively. In 78 (45.3%) patients these parts of the body were not affected. Twenty-seven (15.5%) patients could be assigned to the overlap subtype of cGvHD. The frequency of individual combinations of organ-specific subtypes was visualized in Fig. 1. Most patients (*n* = 110, 63.2%) had severe cGvHD according to global severity score, most of them because of score 3 lung (*n* = 60/110, 54.5%) or skin (*n* = 39/110, 35.5%) involvement. Patients with mild cGvHD had predominantly skin involvement (*n* = 22/34, 64.7%), only eight (23.5%) and six (17.6%) HSCT recipients had liver and GI tract involvement. According to the classification, none of the patients with mild cGvHD had pulmonary manifestations (Fig. 2). The total number of organs involved were median 1 (IQR 1–2), 2.5 (IQR 2–3) and 3 (IQR 2–4) for mild, moderate, and severe cGvHD, respectively.

**Table 1:** Baseline characteristics at ICU admission.

	*n* (%)
Age at time of HSCT, median (IQR)	49 (35.0–57.0)
Male, N (%)	114 (65.5)
Body mass index, median (IQR)	22.7 (20.4–25.3)
Disease, N (%)	
AML	66 (37.9)
MDS	25 (14.4)
ALL	30 (17.2)
MPN/CML	28 (16.1)
Lymphoid diseases	20 (11.5)
Multiple myeloma	4 (2.3)
Aplastic anemia	1 (0.6)
Donor, N (%)^a^	
Related	61 (35.3)
Unrelated	112 (64.7)
HLA constellation, N (%)^a^	
Matched	145 (83.8)
Mismatched	28 (16.2)
Graft source, N (%)^a^	
PBSC	162 (93.6)
Bone marrow	9 (5.2)
Cord blood	2 (1.2)
Conditioning regime, N (%)^a^	
MAC	107 (61.8)
RIC	66 (38.2)
Global severity of cGvHD, N (%)	
Mild	34 (19.5)
Moderate	30 (17.2)
Severe	110 (63.2)
cGvHD organ involvement, N (%)	
Skin	
- any score	132 (76.7)
- score 2	47 (27.3)
- score 3	39 (22.7)
Lung	
- any score	82 (47.1)
- score 2	15 (8.6)
- score 3	60 (34.5)
Liver	
- any score	66 (38.2)
- score 2	15 (8.7)
- score 3	10 (5.8)
GI tract	
- any score	41 (24.1)
- score 2	12 (7.1)
- score 3	12 (7.1)
Eyes	
- any score	51 (29.3)
Mouth	
- any score	46 (26.4)
Fascia	
- any score	19 (10.9)
Serosa	
- any score	4 (2.3)
Year of HSCT, median (IQR)	2011 (2007–2015)
Year of ICU admission, median (IQR)	2014 (2011–2017)
Time from HSCT to ICU admission, median days (IQR)	550 (289–1149)
Time from cGvHD onset to ICU admission, median days (IQR)^b^	357 (106–893)

*ALL* acute lymphoblastic leukemia, *AML* acute myeloid leukemia, *CLL* chronic lymphocytic leukemia, *CML* chronic myeloid leukemia, *cGvHD* chronic Graft-vs-Host disease, *ICU* intensive care unit, *MAC* myeloablative conditioning regimen, *MDS* myelodysplastic syndrome, *MPN* myeloproliferative syndrome, *PBSC* peripheral blood stem cells, *RIC* reduced-intensity conditioning regimen, *HSCT* allogeneic stem cell transplantation.

^a^*Not applicable* in *n* = 1.

^b^*Not applicable* in *n* = 12.

### ICU admission and stay

The median time between HSCT or onset of cGvHD and ICU admission was 550 (IQR 289–1149) and 357 (IQR 106–893) days. Of the 174 patients, five patients were admitted twice. In three cases, these were independent ICU stays with different reasons of admission a time latency of one, 46 and 79 months. One patient was re-admitted after 7 months for re-occurred respiratory failure. Only one patient was re-admitted after 2 months during the same hospitalization.

Main reasons for ICU admission were respiratory failure, which occurred in 122 (70.1%) patients, followed by sepsis in 59 (33.9%) and renal failure in 35 (20.1%) patients. Other reasons were neurological, cardiological and hepatic complications in 18 (10.3%), 16 (9.2%) and 13 (7.5%) patients, respectively. Severe GI problems and pulmonary artery embolism caused ICU admission in 10 (5.7%) and 4 (2.3%) HSCT recipients, respectively. Furthermore, 26 (14.9%) post-operative patients were included, whose surgical procedures were either GvHD-related or associated with permanent use of immunosuppression.

Certain cGvHD subtypes were associated with specific reasons for ICU admission. In patients with pulmonary cGvHD (*n* = 82) respiratory failure led to ICU admission in 70 (85.4%) patients compared to 52 (56.5%) without pulmonary cGvHD (*n* = 92) (*p* < 0.001). ICU admission because of septic shock was less common in this subgroup (19/82 (23.2%) vs. 40/92 (43.5%), *p* = 0.006). There was also a significant association of patients with liver cGvHD (*n* = 66) and ICU admission due to neurological (13/66 (19.7%) vs. 5/107 (4.7%), *p* = 0.003) or hepatic (10/66 (15.2%) vs. 3/107 (2.8%), *p* = 0.006) complications in comparison to patients without liver cGvHD. Skin and GI cGvHD did not correlate with a particular reason for ICU admission (Table S1).

Median duration of ICU stay was 11 (IQR 3–23) days, 8 (IQR 2–19.5) days in ICU survivors and 13 (5.0–30) days in ICU non-survivors (*p* = 0.005). During ICU stay IMV was used in 114 (65.5%) patients, while 20 (11.5%) patients received only NIV. Nearly half of all patients (*n* = 83, 48.5%) required vasopressors during the first 24 h of ICU stay and RRT was necessary in about one quarter of all patients (*n* = 47, 27.0%). Extracorporeal life support devices (ECCO2R, vvECMO, vaECMO) were used in 19 (7, 10, 2, respectively) patients.

### Outcome

After ICU admission, the median follow-up was 68 months (2083 days, IQR 924–3070) with an OS of 28.6% at 3 years. ICU-, hospital- and 1-year survival rates were 57.4%, 51.7% and 36.6%, respectively. The different organ subtypes of cGvHD had no effect on short-term survival, with one exception: Patients with GI tract involvement had lower ICU (39.0% vs. 62.0%, *p* = 0.014) and hospital (34.1% vs. 57.4%, *p* = 0.014)) survival rates compared to patients without GI tract involvement. One-year survival rates for patients with pulmonary, skin, liver, and GI tract cGvHD were 39.1%, 35.5%, 32.9% and 26.3%, respectively (Table S2).

**Fig. 3:**
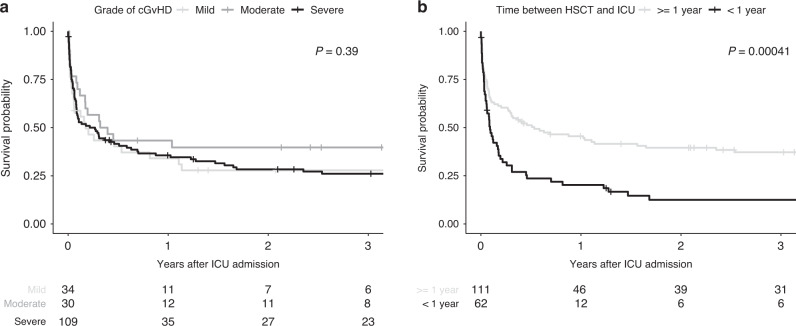
Overall survival of critically ill HSCT recipients with cGvHD. OS depending on (**a**) global severity of cGvHD and (**b**) time between HSCT and ICU admission (within first year of HSCT vs. afterwards).

**Fig. 4:**
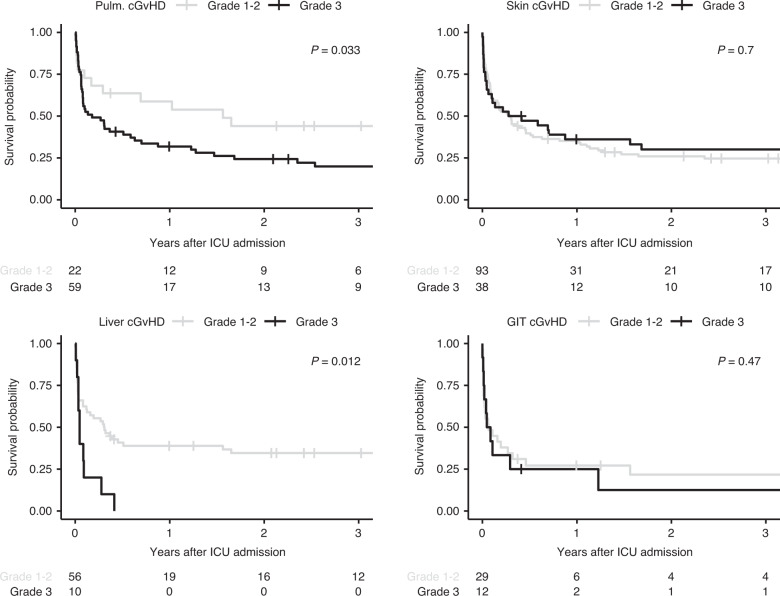
Overall survival of the different cGvHD organ subtypes. Pulmonary cGvHD grade 1–2 vs grade 3; skin cGvHD grade 1-2 vs. grade 3; liver cGvHD grade 1–2 vs grade 3; GIT cGvHD grade 1–2 vs grade 3.

Global severity categories of cGvHD were not significantly associated with ICU- or hospital survival or with OS after ICU admission (Fig. 3a). Isolated analysis of the different organ subgroups also failed to identify an association between cGvHD organ scoring (score 1–2 vs. score 3) and ICU survival (Table S2). HSCT recipients with score 3 cGvHD of the liver had worse hospital survival (*n* = 1/10; 10%) than patients with score 1–2 (*n* = 26/56; 51.8%) and the only patient who survived the hospital stay died within 6 months. Besides score 3 liver cGvHD, OS was reduced in patients with score 3 pulmonary cGvHD compared to patients with pulmonary cGvHD score 1–2 (Fig. 4, *p* = 0.033).

At ICU admission the median prognostic SOFA, SAPS II and APACHE II scores could be calculated for 132, 150 and 144 patients, and were 8 (IQR 4.5–11), 18 (IQR 13.0–22.25) and 40 (IQR 31.25–53.0), respectively. There was a significant difference between ICU-/hospital survivors and non-survivors. Median SOFA score was 6.0 (IQR 3.75–10.25) in hospital survivors compared to 10.0 (IQR 6.0–12.5) in hospital non-survivors (*p* = 0.001). APACHE II Score was 17 (IQR 12–20) in survivors and 20 (IQR 15–24) in non-survivors (*p* = 0.008). None of these scores predicted OS.

Focusing on the ICU treatment intensity, all life sustaining therapies (mechanical ventilation, use of vasopressors, RRT or use of ECCO2R/ECMO) were associated with reduced ICU- and hospital survival. In patients with mechanical ventilation, hospital survival was 33.3% compared to 80.0% in patients with NIV only and 90.0% in patients without ventilation support (*p* < 0.001). Patients with ECCO2R (*n* = 7), vvECMO (*n* = 10) or vaECMO (*n* = 2) support had hospital survival rates of 21.1% compared to 35.8% in mechanical ventilated patients without extracorporeal lung assist (*p* = 0.2904). Use of vasopressors during the first 24 h was associated with reduced hospital survival (42.2% vs. 57.8%; *p* = 0.019), as well as the need for RRT (17.0% vs. 83.0%; *p* < 0.001). Furthermore, all life sustaining therapies, use of vasopressors (32.1% vs. 39.9%, *p* = 0.021), need for mechanical ventilation (23.0% vs. 60.0% (NIV) vs. 64.0% (no MV), *p* < 0.001) and use of RRT (12.8% vs. 45.7%; *p* < 0.001), were associated with reduced 1-year OS rates.

### Respiratory failure in patients with pulmonary cGvHD

In our cohort, IMV or NIV were used in 57 (69.5%) and 12 (14.6%) patients in this subgroup, respectively. Hospital survival rates for HSCT recipients with pulmonary cGvHD were 100% without ventilation support, 75% for use of NIV only and 43.9% for use of IMV (*p* < 0.001). The conditional long-term survival of patients discharged from the ICU was independent of the need for mechanical ventilation (Fig. S1).

### Overlap subtype

We identified 27 HSCT recipients who met the criteria for the overlap subtype. These patients were admitted earlier to ICU after HSCT than those with classic GvHD (263 [IQR 192.5–422.5] vs. 737 [IQR 328–1318] days; *p* < 0.001), and they had lower ICU- (25.9% vs. 63.3%; *p* < 0.001) and hospital survival (22.2% vs. 57.1%; *p* < 0.001) rates. Overlap subtype of cGvHD was associated with worse OS (HR 2.36, 95% CI 1.52–3.66) after ICU admission in univariate analysis.

### Time since HSCT

The time from HSCT to ICU admission (more or less than 1 year) was a significant predictor of long-term survival in patients with cGvHD (3-year OS 37.2% vs. 12.3%; *p* < 0.001 [Fig. 3b]). Patients with early ICU admission after HSCT were slightly older (49 vs. 50 years, *p* = 0.03) and pulmonary cGvHD was less present (32.3% vs. 55.4%, *p* = 0.005) whereas overlap subtype was more frequent (30.6% vs. 7.1%; *p* < 0.001). With a higher SOFA score on day 1 on ICU (9 (IQR 4–12) vs. 8 (IQR 5–11); *p* = 0.005) HSCT patients admitted in their first year post-transplant had a significantly worse ICU- (41.9% vs. 66.1% (*p* = 0.002)) and hospital survival (35.5% vs. 60.7% (*p* = 0.003)) compared to patients whose HSCT transplanted more than 1 year ago.

In a time-dependent multivariate cox regression analysis the need for IMV (HR 1.08 (95% CI 1.02–1.14), *p* = 0.007), RRT (HR 1.73 (95% CI 1.14–2.62); *p* = 0.010) and time from HSCT to ICU admission over 1 year (HR 1.56 (95% CI 1.03–2.39); *p* = 0.035) correlated significantly with reduced OS and were independent prognostic markers. None of the cGvHD subtypes nor cGvHD severity remained significant in multivariate analysis (Table 2).

**Table 2:** Multivariate cox regression for overall survival.

Risk factor	HR	CI 95%	*P* value
HLA mismatch	1.40	0.86–2.29	0.178
Time from HSCT to ICU admission (during/after 1st year of HSCT)	**1.56**	1.03–2.39	**0.035**
GI cGvHD	1.40	0.87–2.72	0.160
cGvHD of other subtypes	0.84	0.52–1.35	0.469
Overlap subtype of cGvHD	1.26	0.68–2.33	0.460
Need for RRT	**1.73**	1.14–2.62	**0.010**
Need for ECLA (ECCO2R/vvECMO/vaECMO: yes/no)	1.54	0.87–2.72	0.137
Need for IMV	**1.08**	1.02–1.14	**0.007**
Need for vasopressors during first 24h	0.97	0.89–1.06	0.543

*cGvHD* chronic Graft-vs-Host Disease, *ECLA* extracorporeal life assist, *GI* gastrointestinal, *HR* hazard ratio, *HSCT* allogeneic stem cell transplantation, *ICU* intensive care unit*, IMV* invasive mechanical ventilation, *RRT* renal replacement therapy.

The bold values mark the risk factors with a *p* value below the significance level of 0.05.

## Discussion

The present study evaluated the outcome of critically ill HSCT recipients with a specific focus on the presence of cGvHD at the time of ICU admission. Despite the patients’ individual morbidity, both short and long-term survival rates were remarkable with a hospital- and 3-year OS of 51.7% and 28.6%, respectively. Life sustaining therapies and time since HSCT but not the global severity of cGvHD were associated with survival. Nevertheless, severe liver cGvHD was associated with reduced hospital survival rate and severe pulmonary cGvHD had a negative impact on long-term survival. However, patients with pulmonary cGvHD and need for IMV long-term survival was a reasonable outcome. Patients admitted during their first year after HSCT had a poorer short- and long-term outcome compared to patients admitted later in course.

Reasons for ICU admission were mainly respiratory failure (69.7%) and septic shock (34.3%), which corresponded to the data already known from studies of critically ill HSCT recipients without a focus on cGvHD [3, 15–17]. These reasons varied depending on the organ systems affected by cGvHD. Patients with pulmonary GvHD were more frequently admitted with respiratory failure, whereas patients with liver dysfunction in the context of cGvHD suffered more often from neurological complications and liver failure.

Furthermore, the use of IMV, vasopressors and RRT was comparable to other critically ill HSCT recipients [3, 15–17], which suggests that there were no specific restrictive therapeutic limitations due to cGvHD in our study centers once patients were selected for intensive care management. To date, there is little data on the frequency of use of extracorporeal lung assist devices in HSCT recipients. Two centers also involved in this study recently analyzed the use of ECCO2R systems and reported their use in 3.4% of all critically ill HSCT recipients during the observation period [18].

The remarkable 1-year OS rate of the whole study population of 36.6% is even higher than the 1-year survival rates reported in recent publications on general HSCT recipients in the ICU [3, 5, 16, 19, 20].

An important finding of our study is, that the global severity of cGvHD did not affect the short- or long-term outcome of critically ill HSCT recipients. Nevertheless, we were able to determine relevant differences for the individual organ subgroups. Patients with score 3 liver cGvHD had a disastrous hospital survival rate of 10% with no long-term perspective, so here the appropriate intensity of ICU treatment should be carefully evaluated. HSCT recipients with score 3 pulmonary cGvHD did not show significantly reduced ICU- or hospital survival compared to pulmonary cGvHD score 1–2, but their survival curve dropped significantly during the first months afterwards effecting long-term survival. These results are in line with the published data from a single-center study in a general cGvHD population [7], demonstrating that severe cGvHD involving the lung, liver, or GI tract was associated with reduced OS, whereas severe cGvHD without involvement of the aforementioned organ systems did not significantly affect long-term survival compared to only mild or moderate cGvHD. In our study, approximately one quarter of all patients with score 3 pulmonary cGvHD survived 2 years or longer, so ICU admission can be offered to this population, at least in a setting of an ICU trial [21] and regardless of the requirement of IMV.

Life sustaining treatments have been previously described as predictor of survival for critical ill HSCT recipients [3, 5, 15–17, 22, 23]. In case of advanced respiratory failure, vvECMO or ECCO2R were used in at least 17 patients in our study with a hospital survival rate of 21.1%. Because the median time between HSCT and ICU admission was more than 1 year in these patients, this result is consistent with the data of Wohlfarth et al [24]. In this multicenter analysis of ECMO use in HSCT recipients time between HSCT and implantation of ECMO (before vs. after 240 days) was reported as a relevant factor of treatment success with hospital survival rates of 4% and 46%, respectively. However, due to the small number of cases, the informative value remains limited and usage of ECCO2R/ECMO should only be discussed as a treatment option for selected patients in experienced centers.

While higher SOFA, SAPS-II, and APACHE-II scores were associated with lower hospital survival rates, they still significantly underestimated mortality in patients with cGvHD. Whereas previous studies in general ICU patients associated a SOFA score of 8 with a mortality of ~20% [25] and a SAPS II score of 18 with a mortality below 5% [12], hospital mortality was 48.3% in our patients with cGvHD.

Some studies reported an association between post-transplant timing and mortality on ICU in HSCT recipients [3, 24]. In our cohort, there might be several reasons for the increased mortality early after transplantation. The patients were older on average and, therefore, likely had more comorbidities. In addition, the higher severity of critical illness reflected by the higher SOFA score may be a decisive factor. Furthermore, the proportion of patients assigned to the “overlap” subgroup was higher. Patients with overlap cGvHD are known for their worse prognosis compared to classical cGvHD in terms of OS and non-relapse mortality [9].

Our study has multifactorial limitations. The retrospective study design resulted in limitations regarding detailed data availability. As already named in the methods, specific subtypes (overlap subtype, hepatic vs. cholestatic liver cGvHD, scoring of cGvHD of the eyes or fascia) have not been ascertainable in detail from the records. Consequently, these aspects of cGvHD could either only be determined retrospectively, as in the case of the overlap subtype, or could not be considered in detail in the statistical analysis. We also do not have sufficient information on the treatment status of cGvHD patients. Intensity, duration, and response to therapy may have influenced patient allocation and outcome.

However, the main limitation of this study is, that it is a solely observational trial of critically ill HSCT recipients with cGvHD, without a control cohort e.g., HSCT recipients without acute or chronic GvHD. To date, there is a lack of published data regarding the prognosis of this patient population. At the beginning of the study period, the data situation for critically ill HSCT recipients in general was even thinner. Consequently, the intensive care allocation of patients and the intensity of their treatment was based on the experiences and judgments of the treating hematologists and intensivists at the respective centers. Universal admission criteria for HSCT recipients were not established in the past and are still not defined in detail for the subgroup of cGvHD patients [26]. All centers included in the study are hospitals with expertize in hematological-oncological intensive care medicine. Over time and from their experience, hematologists and intensivists likely developed better selection criteria for deciding which HSCT recipients might benefit from intensive care treatment and whose prognosis is futile. Intensive care resources for hematological patients may also have played a key role here. Comparative data on the number and course of cGvHD patients who were denied intensive care were not collected for this study. Due to the frequently progressive morbidity and increased mortality of this patient group, discussions about advance care planning and living wills should take place early, especially with patients who have severe, therapy-refractory cGvHD courses. Reflective discussion of the topic and repeated conversations with the treating hematologist about the course and prognosis of cGvHD can lead to better acceptance by the patient and his relatives in the case of medically justified rejection of intensive care procedures. Ideally, the personal will of the patient coincides with the assessment of the physicians through the advanced discussions and thus enables the patient to fully perceive his autonomy. The affiliation to an outpatient palliative care network enables patients to remain in their home environment for as long as possible.

In summary, this study may assist physicians in their decision-making regarding ICU transfers of patients with cGvHD. Our data show an acceptable short- and long-term survival for critically ill patients with cGvHD, except for patients with score 3 liver cGvHD. Remarkably, global severity of cGvHD was not associated with survival. Even for patients with pulmonary cGvHD and respiratory failure requiring ventilation, long-term survival is possible in about a quarter of the cases.

## Supplementary information

Supplemental Material

## Data Availability

The datasets used and/or analyzed during the current study are available from the corresponding author on reasonable request.
