# Supplementary material for: Impact of chronic graft-versus-host-disease on intensive care outcome in allogeneic hematopoietic stem cell recipients
Source: Bone Marrow Transplant. 2022 Dec 10;58(3):303–10. doi: 10.1038/s41409-022-01875-4 (PMC10005928; doi:10.1038/s41409-022-01875-4)
Supplement: Supplementary file 1 — Supplemental Material [file 41409_2022_1875_MOESM1_ESM.docx]

# Supplement

## Table S 1: **Reason for ICU admission depending on cGvHD organ involvement**

| **cGvHD organ involvement** | **Reason for ICU admission** | | | | | | | | | |
| --- | --- | --- | --- | --- | --- | --- | --- | --- | --- | --- |
|  | **ARF** | **Sepsis** | **AKI** | **Cardio** | **Neuro** | **Hepatic** | **GIT** | **Skin** | **PE** | **OP** |
| **Skin (n=132)** | 69.7%  (n=92) | 37.1% (n=49) | 20.5% (n=27) | 9.8% (n=13) | 10.6% (n=14) | 6.8% (n=9) | 5.3% (n=7) | 7.6% (n=10) | 1.5% (n=2) | 15.2% (n=20) |
| **Lung (n=82)** | **85.4%** (n=70)^***^ | **23.2%** (n=19)^**^ | 17.1% (n=14) | 4.9% (n=4) | 9.8% (n=8) | 3.7% (n=3) | 0.0% (n=0) | 3.7% (n=3) | 1.2% (n=1) | 16.6% (n=12) |
| **Liver (n=66)** | 69.7% (n=46) | 42.4% (n=28) | 22.7% (n=15) | 10.6% (n=7) | **19.7%** (n=13)^**^ | **15.2%** (n=10)^**^ | 7.6% (n=5) | 7.6% (n=5) | 1.5% (n=1) | 15.2% (n=10) |
| **GIT (n=41)** | 65.9% (n=27) | 46.3% (n=19) | 22.0% (n=9) | 9.8% (n=4) | 14.6% (n=6) | 4.9% (n=2) | 12.2% (n=5) | 7.3% (n=3) | 0.0% (n=0) | 17.1% (n=7) |

*ARF* acute respiratory failure *AKI* acute kidney injury *Neuro* neurological complications *Hepatic* hepatic complications *GIT* gastrointestinal tract problems *Skin* dermatological problems *PE* pulmonary embolism *OP* operative procedure ** p < 0.01 *** p < 0.001

Table S2: **Clinical outcome by cGvHD organ involvement**

| **cGvHD** | **cGvHD** | **ICU survival** | **Hospital survival** | **12m survival** |
| --- | --- | --- | --- | --- |
| **Skin** | yes (n=132)  *grade 1-2 (n=93)*  *grade 3 (n=39)* | 79 (59.8%)  *56 (60.2%)*  *23 (59.0%)* | 70 (53.0%)  *49 (52.7%)*  *21 (53.8%)* | 43 (35.5%)  *31 (35.2%)*  *12 (36.1%)* |
|  | no (n=40) | 19 (47.5%) | 18 (45.0%) | 15 (39.1%) |
| **Lung** | yes (n=82)  *grade 1-2 (n=22)*  *grade 3 (n=60)* | 51 (62.2%)  *16 (72.7%)*  *35 (58.3%)* | 47 (57.3%)  *16 (72.7%)*  *31 (51.7%)* | 29 (39.1%)  *12 (58.7%)*  *17 (31.8%)* |
|  | no (n=92) | 49 (53.3%) | 43 (46.7%) | 29 (34.5%) |
| **Liver** | yes (n=66)  *grade 1-2 (n=56)*  *grade 3 (n=10)* | 37 (56.1%)  *34 (60.7%)*  *03 (30.0%)* | 30 (45.5%)  *29 (51.8%)*  *01 (10.0%)* | 19 (32.9%)  *19 (38.9%)*  *00 (00.0%)* |
|  | no (n=107) | 62 (57.9%) | 59 (55.1%) | 39 (38.5%) |
| **GIT** | yes (n=41)  *grade 1-2 (n=29)*  *grade 3 (n=12)* | 16 (39.0%)°  *12 (41.4%)*  *04 (33.3%)* | 14 (34.1%)*  10 (34.5%)  04 (33.3%) | 8 (26.3%)  *6 (27.2%)*  *2 (25.0%)* |
|  | no (n=129) | 80 (62.0%)° | 74 (57.4%)* | 50 (40.2%) |

*cGvHD* chronic Graft-vs-Host Disease, *GIT* gastrointestinal tract, *ICU* intensive care unit

° Chi2 p value = 0.010; * Chi2 p value = 0.012

Fig. S1: **Survival with pulmonary cGvHD depending on the ventilation strategies used during ICU stay**
